# Preoperative embolization and craniotomy for the treatment of a tentorial dural arteriovenous fistula

**DOI:** 10.3171/2025.7.FOCVID2597

**Published:** 2025-10-01

**Authors:** Quang Nguyen, Nikolaos Mouchtouris, Yin Hu, Sepideh Amin-Hanjani

**Affiliations:** Department of Neurological Surgery, University Hospitals Cleveland Medical Center/Case Western Reserve University School of Medicine, Cleveland, Ohio

**Keywords:** tentorial dural arteriovenous fistula, craniotomy, clip ligation, endovascular embolization

## Abstract

This is a video presentation of a complex tentorial arteriovenous fistula that required multimodal treatment with preoperative embolization followed by craniotomy for clip ligation with several teaching points. A Cognard type IV fistula has multiple arterial feeders and direct cortical venous drainage with venous ectasia. A multimodal approach was required, consisting of endovascular embolization of occipital feeders followed by tailored craniotomy for clip ligation of the venous outflow of the fistula. The video discusses important nuances for the successful treatment of complex arteriovenous fistulas while minimizing morbidity.

The video can be found here: https://stream.cadmore.media/r10.3171/2025.7.FOCVID2597

## Transcript

This is a video presentation of a complex tentorial AV fistula that required multimodal treatment with preoperative embolization followed by craniotomy for clip ligation.

### 0:30 Patient Presentation.

The patient is a 29-year-old male who presented to an outside hospital with altered mentation. On exam, he was mildly confused, but otherwise neurologically nonfocal. On imaging workup, he was suspected to have an occipital vascular malformation just superior to the tentorium.

### 0:46 Preoperative Imaging.

His postcontrast T1 MRI shows the appearance of a venous varix just medial to the transverse-sigmoid junction. The T2 sequence is shown here redemonstrating a flow void consistent with a dilated vein and showing minimal vasogenic edema.

### 1:04 Preoperative Cerebral Angiogram.

We performed a 6-vessel cerebral angiogram, which showed a dural arteriovenous fistula fed by an enlarged recurrent meningeal artery that originates from the ophthalmic artery as seen on the right internal carotid artery injection. The recurrent meningeal artery feeder supplies the tentorial fistula, which results in cortical venous reflux with significant associated venous ectasia causing a large venous varix. The fistula and draining cortical vein are seen to be immediately adjacent to the location where the vein of Labbé joins the transverse-sigmoid junction, but the flow in the vein of Labbé itself is anterograde into the sinus.

### 1:47 ECA Injection.

The external carotid artery injection showed additional significant supply from the occipital artery with multiple occipital artery branches feeding the fistula, all of which ultimately drained into the cortical vein with the venous ectasia. This retrograde flow ultimately drains into the superior sagittal sinus, as well as into transverse-sigmoid junction through anastomotic flow into the vein of Labbé.

### 2:12 Management Options.

This dural AV fistula is considered high-grade, Borden type III or Cognard type IV due to its direct drainage into cortical veins with venous ectasia and varix formation.^1,2^ High-grade dural AV fistulas with cortical venous drainage have a poor natural history, with a high risk of hemorrhage in the range of 20% annually when presenting with hemorrhage or nonhemorrhagic neurological symptoms, rather than incidentally.^3–5^ Venous ectasia has also been associated with increased hemorrhagic risk. For this patient, given the cortical venous reflux and venous ectasia, we recommended treatment over surveillance. In terms of treatment options, an endovascular approach through the occipital artery, which is an easily accessible, large-caliber vessel with significant feeders to the fistula, was first considered. On the contrary, the recurrent meningeal artery originating from the ophthalmic artery was felt to be higher risk due to the risk of embolization leading to reflux into the ophthalmic artery and the potential associated risk of blindness. For that reason, we prepared for a multimodal approach, first with endovascular embolization to address the occipital feeders and attempt penetration to the drainage point of the fistula if feasible, with a plan to proceed to targeted craniotomy for ligation of the venous fistula and outflow for definitive treatment if needed, whereby the embolization would facilitate the craniotomy by reducing the risk of intraoperative bleeding during the exposure.

### 4:01 Embolization of Occipital Feeders.

For the endovascular treatment, we used Onyx-18 initially to embolize the occipital feeders, followed by the more viscous Onyx-34 to create a plug.

### 4:10 Endovascular Treatment.

Through the superselective runs here and the embolizations that reduce the arterial inflow to the fistula, we were able to gain a better understanding of the architecture of the fistula. The fistulous point is located in the tentorium adjacent to a normally draining vein of Labbé. The draining vein of the fistula exited the tentorium and led to the large venous varix. Interestingly, after embolizing the occipital feeders with some liquid embolic penetration into the fistula, the recurrent meningeal artery began to slow down with decreased flow, as seen on the AP projection of the common carotid run. However, complete penetration of the fistula could not be achieved and therefore we proceeded with the surgical plan.

### 4:52 Operative Setup.

Our goal for surgery was then to perform a tailored craniotomy centered right over the draining vein of the fistula, just superior to the tentorium, and to ligate the vein at its site of egress from the dura. For this location, we positioned supine with the head turned and the shoulder elevated to reduce tension on the neck and reduce jugular venous outflow obstruction. We obtained a CTA of the head after the embolization for neuronavigation. We used a radiolucent skull clamp and prepped and draped accordingly for an intraoperative cerebral angiogram through femoral access.

### 5:31 Postembolization CTA.

The postembolization CTA shows the location of the venous varix in relation to the tentorium, transverse sinus, and the Onyx embolic material, making it easier to perform a small craniotomy centered over the pathology. The presence of the embolic material also can also help navigate intraoperatively and confirm the accuracy of the registration.

### 5:52 Exposure.

This is our intraoperative view from the surgical microscope. The patient is in supine head-turned position with the occipital lobe toward the bottom of the screen and the tentorium at the top of the screen. The vein of Labbé is seen on the left of the screen and the venous varix is toward the right of the screen. We start by identifying the tentorium, then dissecting around the venous varix and tracing the vein proximally toward the tentorium in order to isolate the fistula drainage point. Once we had dissected enough to visualize the fistula, we performed an ICG run, which confirmed the early draining vein feeding the venous varix before the normal capillaries of the parenchyma are filling followed by the vein of Labbé filling.

### 6:35 Clip Ligation of the Fistulous Point.

We continued to dissect around the draining vein that exits the tentorium and thoroughly coagulate the adjacent dura at the location of the tentorium, with care to avoid the insertion site of the vein of Labbé. We created space around the draining vein so that we could circumferentially coagulate and ligate. As we coagulated the draining vein, the varix softened and turned more blue in color. Lastly, we placed a permanent titanium clip as proximal on the draining vein as possible where it emanated from the tentorium and then cut the draining vein. We also placed the clip closer to the venous varix as a marker for the subsequent angiogram.

### 7:12 Postligation ICG.

Once completed, we performed a postligation ICG run to confirm that there was no early venous drainage, that the varix did not fill, and that the vein of Labbé was filling appropriately after the capillary phase. We also placed a small piece of Gore-Tex material between the tentorial dura and brain surface to act as a physical barrier, with the intent of reducing the risk of fistula recurrence.

### 7:38 Intraoperative Angiogram.

We performed an intraoperative angiogram, which shows the successful obliteration of the dural AV fistula with no signs of cortical venous reflux or the venous varix, and the vein of Labbé still filling appropriately. The occipital artery is opacified until the venous phase only because it is occluded distally with the Onyx embolic material and the contrast is slow to wash out.

### 8:01 Oblique Run.

Here is an oblique view to better visualize the ligated fistula in relation to the vein of Labbé. The clips are highlighted here in this run. We favor placement of small clips in the location of fistula obliteration in addition to the coagulation that is performed to obliterate the fistula as these clips can serve as identifiable markers on postoperative imaging and help to orient in the event of a residual or recurrent fistula.

### 8:25 Postoperative Imaging.

Postoperative imaging is shown here with no signs of venous infarct or hemorrhage. With the help of neuronavigation, we were able to make a small supratentorial craniotomy and address the pathology effectively.

### 8:37 Postoperative Course.

The patient did well postoperatively and remained neurologically intact and was able to be discharged the following day. In general, we recommend performing follow-up angiography 6 to 12 months post–fistula obliteration to ensure no evidence of recurrence, but in this case the patient declined and we are following him clinically. He has remained neurologically intact without any symptoms at his 2-year follow-up.

### 9:01 Discussion.

There are several teaching points to consider for this case. Dural AV fistulas with multiple feeders can appear to be an overwhelming pathology at first glance but are manageable with thorough planning. Endovascular embolization can be a definitive treatment or, as in this case, an adjunctive treatment even if surgery is required.^6^ In this case, preoperative embolization decreased the number of feeders and reduced the flow through the fistula, enabling better visualization and understanding of the pathology, but also facilitating a safer craniotomy. In terms of the surgery, the primary goal should be to disconnect the cortical venous drainage from the arterial inflow and fistula, not necessarily resection of the fistula itself.^7,8^ In this case, that was achieved by simply ligating the draining vein as it emerged from the fistula just above the tentorium. There was no need to incise the tentorium and resect the intradural fistula, which would have posed additional risk of disrupting the anterograde drainage of Labbé into the transverse-sigmoid sinus. Lastly, in our case, the vein of Labbé was large and in close proximity to the fistula but was unrelated to the fistula itself and draining physiologically. It is therefore important to carefully study the preoperative films as well as perform an ICG run intraoperatively to distinguish early venous drainage from physiological veins and only ligate the fistulous outflow.

## Disclosures

The authors report no conflict of interest concerning the materials or methods used in this study or the findings specified in this publication.

## Author Contributions

Primary surgeon: Amin-Hanjani. Assistant surgeon: Hu. Editing and drafting the video and abstract: Nguyen, Mouchtouris. Critically revising the work: Amin-Hanjani, Nguyen, Mouchtouris, Hu. Reviewed submitted version of the work: Amin-Hanjani, Nguyen. Approved the final version of the work on behalf of all authors: Amin-Hanjani. Supervision: Amin-Hanjani.

## Supplemental Information

### Patient Informed Consent

The necessary patient informed consent was obtained in this study.
